# Supplementary material for: RNA Sequencing Analysis Reveals Divergent Adaptive Response to Hypo- and Hyper-Salinity in Greater Amberjack (Seriola dumerili) Juveniles
Source: Animals (Basel). 2022 Jan 29;12(3):327. doi: 10.3390/ani12030327 (PMC8833429; doi:10.3390/ani12030327)
Supplement: Supplementary file 1 [file animals-12-00327-s001.zip › Approval of Animal Use Protocol.pdf]

广东海洋大学水产学院实验动物福利伦理审查同意书

Approval of Animal Use Protocol, IACUC, GDOU

批准编号 Approval No.	**GDOU-IACUC-2021-A1220**

本动物实验方案经过广东海洋大学水产学院实验动物伦理委员会审核,符合动物保护、动物福利和伦理原则,符合国家实验动物福利伦理的相关规定。

The animal use protocol listed below has been reviewed and approved by the Institutional Animal Care and Use Committee (IACUC), Fisheries College, Guangdong Ocean University.

<table>
<tr><td rowspan="2">实验名称
Protocol Title</td><td colspan="5">盐度胁迫下高体鰤鳃组织转录组分析</td></tr>
<tr><td colspan="5">RNA-seq analysis reveals divergent adaptive response to hypo- and hyper-salinity in greater amberjack (Seriola dumerili) juveniles</td></tr>
<tr><td>实验申请人
Applicant</td><td>彭宇濠
Peng Yuhao</td><td>职称/学位
Title/Degree</td><td>硕士研究生
Master degree candidate</td><td>邮箱
Email</td><td>pengyuhaopyh@163.com</td></tr>
<tr><td>实验负责人
Principle Investigator (PI)</td><td>石红娟
Shi Hongjuan</td><td>职称/学位
Title/Degree</td><td>讲师
Lecture</td><td>邮箱
Email</td><td>shihj@gdou.edu.cn</td></tr>
<tr><td>院系(部门)
Department</td><td colspan="2">水产学院
Fisheries college</td><td colspan="2">申请日期
Application date</td><td>2020/12/1</td></tr>
<tr><td>动物种系
Species or Strains</td><td colspan="2">高体鰤
Seriola dumerili</td><td colspan="2">动物数量 Quantity</td><td>200</td></tr>
<tr><td>计划执行时间
Period of Protocol</td><td colspan="2">2020 年 12 月 1 日至
2022 年 12 月 31 日</td><td colspan="2">实验动物使用许可证
License No. of Laboratory Animal Facility</td><td>水产学院
Fisheries college</td></tr>
<tr><td>审查意见
Results of Review</td><td colspan="5">【 √ 】符合动物福利伦理要求，同意实验 Agree
【 　 】调整方案后，可进行实验 Agree after modification
【 　 】不同意 Disagree</td></tr>
<tr><td>批准人
Approver</td><td colspan="2">李广丽 Guangli LI</td><td colspan="2">日期 Date</td><td>2021.12.20</td></tr>
</table>

广东海洋大学水产学院

IACUC, Fisheries college of Guangdong Ocean University
