# Supplementary material for: RNA Sequencing Analysis Reveals Divergent Adaptive Response to Hypo- and Hyper-Salinity in Greater Amberjack (Seriola dumerili) Juveniles
Source: Animals (Basel). 2022 Jan 29;12(3):327. doi: 10.3390/ani12030327 (PMC8833429; doi:10.3390/ani12030327)
Supplement: Supplementary file 1 [file animals-12-00327-s001.zip › Certification of English editing.pdf]

MogoEdit

CERTIFICATE OF ENGLISH EDITING

This is to certify that the manuscript entitled

RNA-seq analysis reveals divergent adaptive response to hypo- and hyper-salinity in greater amberjack (Seriola dumerili) juveniles

commissioned to us has been carefully edited by a native English-speaking editor of MogoEdit, and the grammar, spelling, and punctuation have been verified and corrected where needed. Based on this review, we believe that the language in this paper meets academic journal requirements. Please contact us with any questions.

MOGOEDIT
MOGO INTERNET TECHNOLOGY CO., LTD.

Gang Zhang

Dr. Gang Zhang
Founder & CEO of MogoEdit

Date of Issue
December 14, 2021

Disclaimer: The changes in the document may be accepted or rejected by the authors in their sole discretion after our editing. However, MogoEdit is not responsible for revisions made to the document after our edit on **December 14, 2021**.

MogoEdit is a professional English editing company who provides English language editing, translation, and publication support services to individuals and corporate customers worldwide. As a company invested by the affiliate fund of Chinese Academy of Science, MogoEdit is one of the leading language editing service providers in China, whose clients come from more than 1000 universities and research institutes.
MogoEdit Website: http://en.mogoedit.com/
500+ native English editors: http://en.mogoedit.com/editors

Mogo
make the world flatter
