# Supplementary material for: RNA Sequencing Analysis Reveals Divergent Adaptive Response to Hypo- and Hyper-Salinity in Greater Amberjack (Seriola dumerili) Juveniles
Source: Animals (Basel). 2022 Jan 29;12(3):327. doi: 10.3390/ani12030327 (PMC8833429; doi:10.3390/ani12030327)
Supplement: Supplementary file 1 [file animals-12-00327-s001.zip › Supplementary (Table S1& Figure S1).pdf]

Table S1. Primer sequences used in this study

Gene	Primer name	Primer sequence (5′-3′)	Purpose
β-actin	*β-actin*-F	TGATGAAGCCCAGAGCAAGAG	qPCR
	β-actin-R	CGTTGTAGAAGGTGTGATGCCA	
ebp	*ebp*-F	GATTCCCACCTATGTTGCCAA	
	ebp-R	CCAGACGCCTCCATGTTCC	
edar	*edar*-F	GGAGAACCGACCCAGAAACC	
	edar-R	GACCCTGTCCATTGGAACCT	
msmo1	*msmo1*-F	AAATACATCCACAAAGTCCACCA	
	msmo1-R	GTCTCCAGCAGGCGGAAAGC	
nsdhl	*nsdhl*-F	TGTCCGACCGAGCAGTAAGC	
	nsdhl-R	TGGCAGCAGAGCCTGTTTGT	
sqle	*sqle*-F	CACCGACCACCACCTCTTCT	
	sqle-R	CACTTGCCCTTTAGCCTTCA	
lss	*lss*-F	TCCGAGACGCCCATCAGTTT	
	lss-R	TCCAGCCGCAGTCACGAGTA	
ogdh	*ogdh*-F	TCGGCTCAATGTCCTGGCTAA	
	ogdh-R	GCTTCAAGGTGGGACGGGTT	
wnt4	*wnt4*-F	GATTATTTACGCCCGTCTCC	
	wnt4-R	TTCAGCAAAGCATCCTCCAC	
slc25a48	*slc25a48*-F	CCTCACCATCTACAGGAAGGAAAC	
	slc25a48-R	TCTGTGTGTTACTGAAGAAGCCAAA	

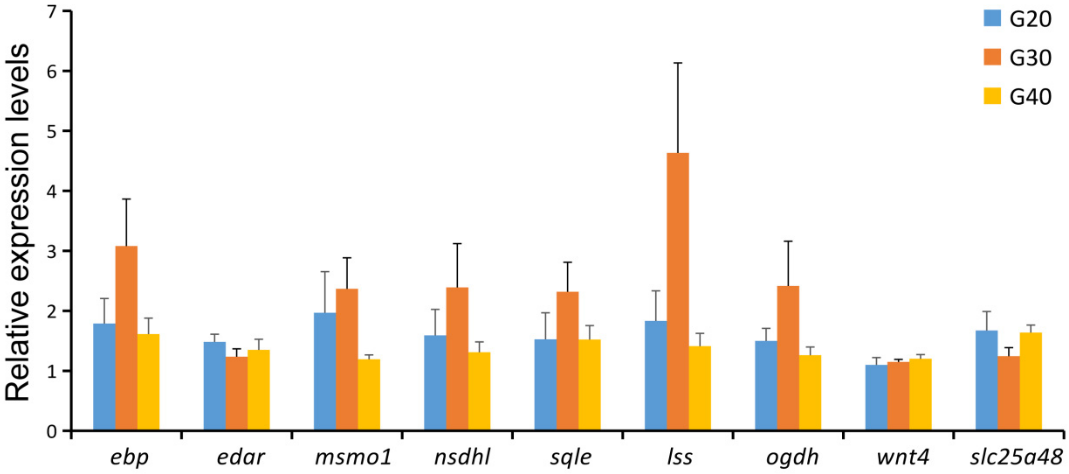

Figure S1. Relative expression levels of different genes in RT-PCR (samples taken on the 15th day).
