# Supplementary figures and images for: RNA Sequencing Analysis Reveals Divergent Adaptive Response to Hypo- and Hyper-Salinity in Greater Amberjack (Seriola dumerili) Juveniles
Source: Animals (Basel). 2022 Jan 29;12(3):327. doi: 10.3390/ani12030327 (PMC8833429; doi:10.3390/ani12030327)

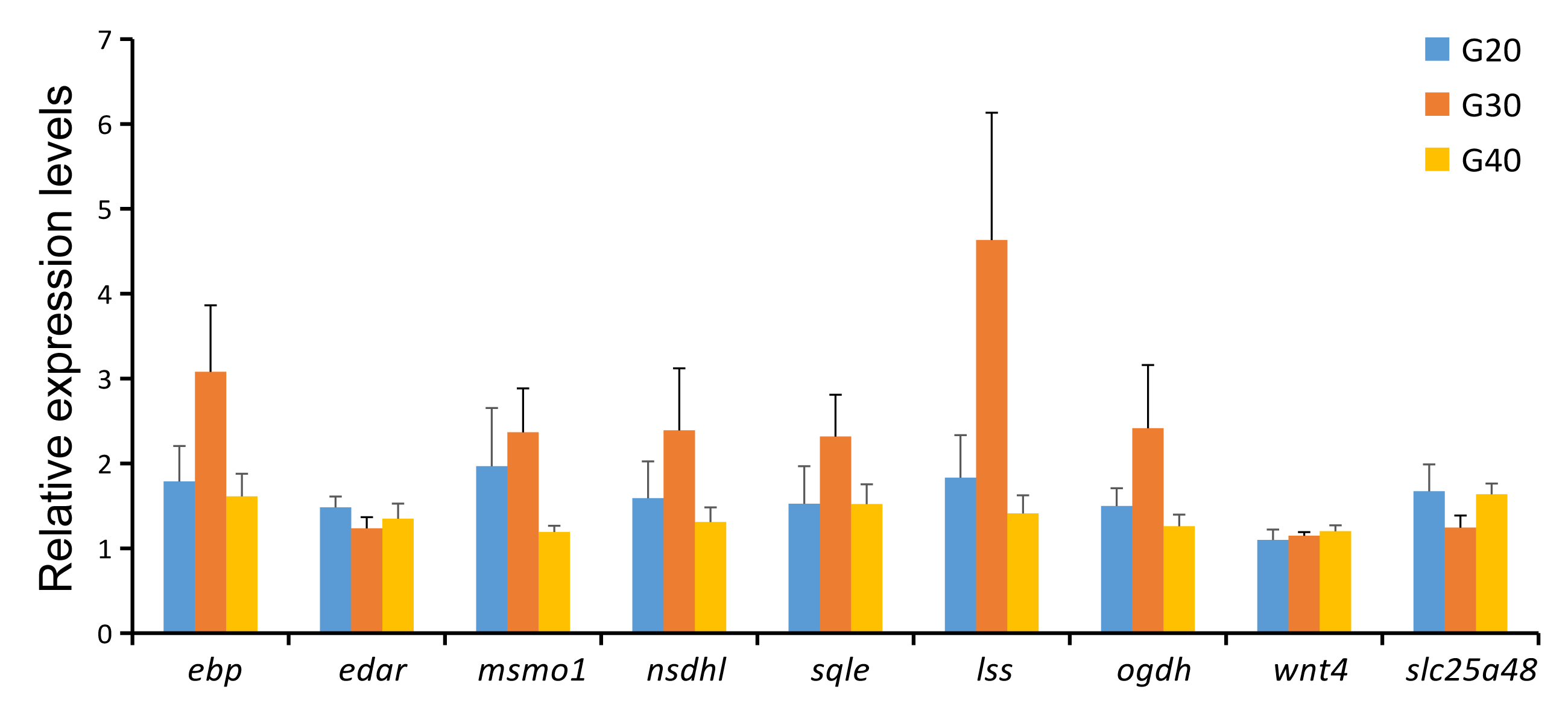

Supplement: Supplementary file 1 [file animals-12-00327-s001.zip › Figure S1.tif]
